# New Appliances and Things Medical

**Published:** 1894-05-26

**Authors:** 


### NEW APPLIANCES AND THINGS MEDICAL.

[All preparations, appliances, novelties, &c., of which a notice is desired, should be sent for the Editor, to care of The Manager, 428, Strand, London, W.C.]

### A NEW FORM OF FILTER.

Being invited to attend at a demonstration of the action of the "Nibestos" filters at 126, Charing Cross Road, our representative called at the offices of the company on Thursday last. A considerable number of filters were in full action, and we were enabled by a variety of experiments to satisfy ourselves that the practical results obtainable from the Nibestos filters are such as have never before been obtained from any other form of filter. The success of these filters depends on the satisfactory production of an asbestos tissue or cloth, which is of such exquisitely fine texture, that such minute particulate matter as ultramarine or bacteria such as occur in water, are quite unable to pass through the meshes. The asbestos tissue, however, is friable and easily damaged, and hence it is ingeniously protected between two layers of stout asbestos cloth which are able to resist ordinary degrees of pressure. The rate of filtration is highly satisfactory, and may be varied according to requirements by increasing the pressure under which the water filters, or by increasing the area of the filtering surface. Quite justly the manufacturers condemn the practice of filtering under high pressure, since, if the pressure be high enough, solid matter may be forced through any medium, however fine be the texture. Under ordinary circumstances the asbestos tissue, or "nibestos," as the manufacturers call it, must be occasionally replaced by a new disc, since, in common with all other filtering media, it must, after a time, become befouled. The method of changing the disc is easy and the cost small, so that there is no excuse for negligence in this respect. Amongst the patents taken out by the firm are included some very ingenious devices for aerating the water, and for cleaning the filtering surface by an automatic mechanism. This latter device depends on a principle which is so designed that the water for ordinary domestic use, and which does not require filtering, thoroughly flushes the surface of the filter pad, and thus removes the precipitate which has been removed from the filtrate, and which is to be used for drinking purposes. The methods are so ingenious and practicable that they are likely to prove as great a success in this country as they have already done in America.

### PYROZONE SOLUTIONS.

(McKessor and Robbins, 91, Fulton Street, New York, U.S.A.)

The pyrozone solutions, as forwarded to our offices by the above firm, were found on analysis to be accurate, 3 per cent. aqueous, 5 per cent. and 25 per cent. ethereal solutions of $H_2 O_2$. The value of having preparations of known and reliable percentages of this substance is a great desideratum for all those who may from time to time prescibe this powerful antiseptic. The uses to which the various percentages may be applied are numerous and varied. For instance the 3 per cent. aqueous solution may be used internally or externally, in the former case for indigestion combined with fermentative processes in the stomach, for ulcers in the stomach, and gastritis; and in the latter case as an antiseptic lotion for washing wounds or sinuses. The 5 per cent. ethereal solution is a powerful antiseptic, the rapid evaporation of the ether leaving the pyrozone in ever increasing strength after application. It may be applied to the unhealthy surfaces in atrophic rhinitis, ulceration of the cutaneous surfaces or mucous membranes, and in diphtheria. The 25 per cent. solution may be used for the same purposes as the above, only it must be remembered that the strength is five times as great, and, moreover, has a powerful caustic action, and in this respect it is a very efficacious application to specific sores, the crusts of contagious impetigo, and to patches of ringworm. In dental practice there can be no doubt that pyrozone has a great future before it, both in the treatment of alveolar abscesses in strong solution, and as an ordinary mouth wash in a weak aqueous solution. The glass atomizer for applying pyrozone as a spray, and the glass syringes and other appliances used in the handling of this powerful destroyer of organic substances, are distinct advances on the older forms.

### BYRRH.

(Violet Frères, 68, Cheapside, E.C.)

Byrrh is an appetising and tonic wine which has acquired on the Continent a very wide-spread popularity. It is described by the manufacturer as being made from pure, natural, and generous wine, the principal bitters employed in its preparation being Chinchona barks. Were it not for the rich red colour of the tonic, one would imagine that one was drinking sherry and bitters, and hence it may be readily understood that Byrrh is by no means a disagreeable beverage. As an appetiser and pick-me-up Byrrh is certainly second to none of the ordinary mediums for these purposes. Our analysis shows that it is an alcoholic drink containing about 7·98 per cent. of absolute alcohol by weight, or 9·2 per cent. by volume. Its strongly stimulant effect, combined with the bitter taste, suggested the idea that some of the extraction of the seeds of strychnos nux vomica, might be employed in the manufacture; our analysis however showed this suspicion to be unfounded. We believe that Byrrh is a powerful tonic and stimulant, but its application should not be abused, as it is an alcoholic beverage of nearly the same strength as ordinary port wine.

